# HNF4α as a Master Regulator of Epigenetic Dynamics in Epithelial Cells

**DOI:** 10.3390/genes17010041

**Published:** 2025-12-31

**Authors:** Laura Amicone, Carla Cicchini, Alessandra Marchetti

**Affiliations:** Department of Molecular Medicine, Sapienza University of Rome, Viale Regina Elena 324, 00161 Rome, Italy

**Keywords:** master factor, epigenetic regulation, chromatin remodeling, transcriptional networks, ncRNA, EMT/MET

## Abstract

Hepatocyte nuclear factor 4 α (HNF4α) is a master transcriptional regulator essential for the maintenance of epithelial cell identity and function. Beyond its well-established role in controlling metabolic and differentiation programs, recent evidence highlights HNF4α as a key determinant of epithelial epigenetic reprogramming. Through direct interaction with chromatin modifiers and pioneer factors, HNF4α contributes to the establishment, maintenance, and dynamically reshaping of epithelial-specific transcriptional programs at epigenetic level. In this review, we summarize current knowledge on how HNF4α shapes chromatin organization by recruiting chromatin modifiers, modulating nucleosome positioning and regulating chromatin loop formation, thus directing tissue-specific gene expression. We also examine its direct regulation of epigenetic modifiers, as well as of epi-miRNAs and epi-lncRNAs, underscoring its role in coordinating chromatin remodeling with transcriptional networks. Finally, we address how dynamic HNF4α occupancy and activity influence context-dependent transcriptional outputs, and how disease-related alterations of its expression and function can contribute to epithelial dysfunction. Understanding the epigenetic functions of HNF4α provides new insights into epithelial biology and reveals potential therapeutic opportunities for restoring epithelial homeostasis in disease contexts.

## 1. Introduction

Hepatocyte nuclear factor 4α (HNF4α) is a liver-enriched member of the orphan nuclear receptor superfamily that mainly acts as a transcriptional activator but also as a repressor.

It is expressed in a variety of epithelial tissues, including liver, kidney, pancreas and gut where it plays a crucial role during embryonic development as well as in the maintenance of epithelial identity and in the execution of differentiative programs [1,2,3,4].

The pivotal function of HNF4α in development is underscored by the embryonic lethality observed in liver-specific knockout mice. Deletion of HNF4α in the fetal liver results in the severe disorganization of hepatic architecture and impaired hepatocyte differentiation, accompanied by the acquisition of mesenchymal features [5]. Otherwise, conditional depletion of HNF4α in adult animals primarily leads to metabolic disorders, particularly affecting lipid homeostasis [6].

Conversely, ectopic expression of HNF4α in fibroblasts has been shown to induce a mesenchymal-to-epithelial transition (MET) [5] and in poorly differentiated and invasive hepatocellular carcinoma (HCC) cells to restore an epithelial differentiated phenotype, thereby reducing both proliferation and invasiveness [7,8,9]. Together with evidence that HNF4α regulates more than two thousand genes, as demonstrated by global transcriptomic analyses following its silencing in cultured hepatocytes or in animal models [10], these findings establish HNF4α as a master regulator of hepatocyte identity and a tumor suppressor.

Accumulating evidence over recent years indicates that HNF4α exerts its regulatory functions primarily through epigenetic mechanisms. It operates directly, by binding to the chromatin of its target genes to modulate the DNA accessibility or by recruiting chromatin-modifying complexes, and indirectly, by controlling the expression of various epigenetic regulators, including chromatin modifiers and non-coding RNAs (ncRNAs).

In this review, we will discuss the role of HNF4α in the epigenetic regulation of gene expression, emphasizing its pivotal contribution to dynamic cellular processes and to the long-term maintenance of cell type-specific identity.

## 2. HNF4α as Both Pioneer-like and Bookmarking Transcription Factor

Following the identification of the HNF4α-specific DNA binding motif [11], genome-wide analyses have delineated its extensive binding profile and predicted thousands of target genes [12,13]. Subsequent ChIP-seq studies in hepatocytes and other epithelial cells confirmed that HNF4α occupies many genomic loci, while RNA-seq analyses of HNF4α knockout or overexpression models provided complementary insights into its transcriptional outputs [1,4,10]. Studies of chromatin occupancy revealed that only a subset of HNF4α-bound regions is transcriptionally active, with functional binding depending on the presence of specific activating histone modifications [14]. Moreover, HNF4α chromatin occupancy and activation of HNF4α-bound promoters are both dynamic and context-dependent, varying according to cell type, differentiation status, and regulatory pathway activity [4,14,15].

Emerging evidence indicates that HNF4α can exert regulatory effects even prior to the establishment of canonical epigenetic marks. In this capacity, HNF4α functions as a bookmarking transcription factor, a specific class of regulatory factors acting downstream of pioneer factors to maintain or propagate an open, transcriptionally competent chromatin state, thereby “marking” genomic loci for efficient future activation [16].

During liver development, HNF4α displays a bookmarking behavior: integrated analyses of chromatin occupancy, histone modifications, and gene expression demonstrated that it binds dynamically to regulatory regions immediately after pioneer factors, driving the establishment of active promoter configurations [17]. From embryonic day 15.5 onward, the binding results in transcriptional activation but only when associated with activating histone marks. Consequently, HNF4α sustains and propagates an open chromatin environment without initiating decondensation itself, contributing to transcriptional competence and proper gene activation during hepatic differentiation.

Recent evidence suggests that HNF4α can display not only bookmarking activity but also pioneer-like properties, although it is not considered a canonical pioneer factor (it does not bind nucleosomes, lacks DNA scanning activity, and does not occupy regions lacking H3K4me1/H3K27ac) [18]. Quantitative analyses using the “pioneer activity index” [19] indicate that HNF4α can bind a subset of relatively inaccessible genomic regions and may facilitate the initial establishment of chromatin accessibility in certain developmental or genomic contexts. However, this pioneer-like activity appears to be highly context-dependent (e.g., following ectopic expression of HNF4α in mesenchymal cells) [20] and substantially less generalized than that of classical pioneer factors [21] such as FOXA1/2 proteins [22]. During early hepatic development, HNF4α can stabilize or increase accessibility at selected regulatory regions, following or cooperating with pioneer factors, while in differentiated hepatocytes, it primarily functions as a bookmarking transcription factor, maintaining an open chromatin state and marking regulatory regions for subsequent transcriptional activation. Although FOXA binding to DNA precedes that of HNF4α during liver development [23], recent data shows that both factors can independently activate silent genes, indicating the pioneer activity as a quantitative and context-dependent property rather than a fixed functional category [24].

Overall, these findings highlight the dual, context-dependent roles of HNF4α, as pioneer-like and bookmarking factor, whose functions range from the stabilization and boost of transcriptional competence to the priming of chromatin accessibility depending on chromatin state and interactions with other regulatory factors.

## 3. HNF4α as a Master Regulator of Gene Expression

HNF4α plays a central role in controlling tissue-specific transcriptional programs, particularly within liver, pancreas, intestine, and kidney lineages [3,25,26,27] by coordinating epithelial and metabolic gene networks.

In particular, in a pioneering study by Odom et al. [25] based on ChIP-chip analysis, HNF4α was shown to bind directly to almost half of the actively transcribed genes in hepatocytes and islet beta cells, thus emerging as a central element of the hepato-pancreatic transcriptional network. Moreover, as reported by the recent review of Vemuri et al. [27], convergent evidence from integrative genomic, genetic, and functional studies (RNA-seq, ATAC-seq, conditional knockouts, organoids, ChIP-seq) further underlined the central role of HNF4α and of its paralog HNF4γ in the control of the intestinal transcriptional program driving several enterocyte functions, including apical absorption, metabolism, and proliferation vs. differentiation. Regarding the kidney, HNF4α plays an important role in the development and differentiation of the proximal tubular epithelium. This function has been demonstrated in KO mice, where the loss of relevant membrane transporters and, more generally, the functional impairment and structural abnormalities of the brush border led to the development of a Fanconi-like syndrome, characterized by severe deficit of the renal reabsorption rate [3,28].

In liver, this transcriptional factor fulfills the defining criteria of a master regulator because it acts on a large number of genes, responsible for liver development and hepatocyte differentiation during embryogenesis and fetal life, and for the maintenance of hepatocyte identity and the execution of complex liver functions in adults, including lipid metabolism, gluconeogenesis, drug metabolism and detoxification [26,29]. Furthermore, HNF4α directly regulates other factors, such as HNF1α HNF6, FOXA1/2, and C/EBPα, establishing a hierarchical and complex cross-regulatory transcriptional network that enforces specific gene expression in hepatocytes and pancreatic islets [25,30].

The evidence supporting HNF4α designation as a master regulator arises from loss-of-function and reprogramming studies. Conditional deletion of HNF4α in hepatocytes or enterocytes leads to a widespread downregulation of tissue-specific genes and loss of epithelial identity, highlighting its essential role in maintaining differentiated states [5,31]. Conversely, its forced expression has been shown to reprogram fibroblasts into hepatocyte-like cells, further confirming its instructive role in specifying hepatic fate [5]. Moreover, HNF4α in combination with GATA4 and FOXA3 can directly reprogram somatic cells into fully differentiated hepatocytes (the so-called induced hepatocytes, iHeps) widely used instead of primary hepatocytes in experimental approaches of liver regeneration and hepatic tissue engineering [32,33].

The extensive reprogramming of gene expression induced by forced expression of HNF4α has relevance in tumoral cells where it can act as tumor suppressor. In fact, exogenous HNF4α can antagonize the epithelial-to-mesenchymal transition (EMT) process in dedifferentiated and invasive HCC cell lines by inducing the reverse process of mesenchymal-to-epithelial transition (MET) [9]. The ectopic expression of HNF4α reduces invasiveness, migration and ability to metastasize in in vivo models through the downregulation of the EMT master genes Snail, Twist and Slug while it induces epithelial and metabolic genes [9,34,35].

Mechanistically, the HNF4α function as a master factor of epithelial differentiation and metabolic homeostasis lies in its ability to integrate transcriptional and epigenetic regulation. HNF4α, in fact, binds to DR1-type response elements in cooperation with transcriptional cofactors and chromatin modifiers. The recruitment of chromatin remodelers and histone-modifying enzymes maintains open chromatin at active tissue-specific enhancers, particularly in the liver, where HNF4α sustains a specific active epigenetic landscape [36].

## 4. HNF4 Shapes Epigenetic Landscapes to Control Cell-Specific Transcriptional Programs

The first evidence of HNF4α’s influence on chromatin state dates back several years. In 1999, overexpression of HNF4α in fibroblasts was shown to alter DNase I sensitivity at the Serpin gene cluster on 14q32.1, affecting transcription of genes encoding α1-antitrypsin and corticosteroid-binding globulin [37]. Since then, several studies have documented the widespread binding of HNF4α to genomic cis-regulatory modules (CRMs) across multiple adult endodermal organs, including liver, kidney, intestine, and pancreas [26]. HNF4α is recruited to both promoters and enhancer regions, where it orchestrates gene expression programs that maintain epithelial identity and support tissue-specific cellular functions, such as those of hepatocytes, enterocytes, and renal epithelial cells [3,31,38,39,40].

Although a substantial overlap exists in HNF4α-occupied chromatin regions across different epithelial tissues, the functional significance of its binding becomes apparent only when chromatin modifications and transcriptomic data were integrated. For instance, comparative ChIP-seq analyses of HNF4α binding in intestinal and renal genomes revealed extensive shared occupancy (~11,000 sites), with only a small subset of regions showing differential recruitment [1]. Significantly, integrated analyses combining chromatin accessibility and transcriptomics in intestinal cells demonstrated that these differentially HNF4α-bound regions correspond to tissue-specific gene expression, highlighting the context-dependent regulatory role of HNF4α [40].

During embryonic development, coordinated changes in cell identity are driven by dynamic remodeling of transcriptional networks based on HNF4α redistribution and/or recruitment of additional transcriptional factors and epigenetic modifiers.

HNF4α has been described as an essential component of the transcription factor network shift that drives the embryonic-to-fetal transition in the intestine. ATAC-seq analyses revealed that accessible chromatin regions change dynamically during intestinal development from CDX2 motifs in embryonic stages to motifs for HNF4α in fetal stages (>E16.5). Genetic inactivation of *Hnf4α* and its paralog *Hnf4g* demonstrated that HNF4 factors are redundantly required for fetal intestinal maturation. CDX2 directly binds and activates *Hnf4* loci, increasing HNF4α expression during the fetal stages. Subsequently, HNF4α and CDX2 co-occupy shared genomic regulatory regions, to promote chromatin accessibility and the transcriptional programs necessary for intestinal differentiation [26]. Moreover, Vemuri et al. [41] demonstrate the critical role of HNF4α in orchestrating dynamic changes in RNA polymerase II activity during intestinal differentiation. Loss of HNF4α function disrupts Pol II occupancy at differentiation-associated genes while enhancing transcription of genes linked to proliferation and stress responses, underscoring HNF4α’s central role in maintaining the transcriptional programs that sustain intestinal identity. Furthermore, HNF4α interacts with chromatin remodelers such as the SWI/SNF complex (including SMARCA4, SMARCE1, and SMARCD2) and with cohesin components like SMC1A and SMC3 [41]. These interactions suggest that HNF4α promotes chromatin accessibility and facilitates the formation of enhancer–promoter loops, thereby establishing a permissive environment for Pol II recruitment and stable occupancy at differentiation genes.

Regarding liver development, in a murine model, Alder et al. [15] identified thousands of enhancers bound by HNF4α and FOXA2 in a differentiation-dependent manner. These enhancers undergo chromatin remodeling, rather than simple Polycomb-mediated repression, consistent with the role of HNF4α and FOXA proteins as pioneer factors capable of binding compacted nucleosomes. Enhancers active exclusively during embryonic stages respond to the transcription factor TEAD2 and its coactivator YAP1, key components of the Hippo signaling pathway [42], which modulate hepatocyte differentiation by influencing HNF4α and FOXA2 interactions with “temporal enhancers”. These enhancers coordinate the expression of genes essential for hepatic lineage specification and the proliferation of liver progenitor cells. As development proceeds, activation of the Hippo pathway induces YAP1 phosphorylation and nuclear exclusion, resulting in reduced TEAD2 activity. Consequently, embryonic enhancers are silenced or replaced by other enhancers active in mature hepatocytes [15]. In the temporal enhancer switching that occurs during liver development, epigenetic modifications appear to play a pivotal dual role: firstly, by priming enhancers while they are still inactive, and then by stabilizing the chromatin conformation that becomes accessible to subsequent activating modifications. HNF4α has been demonstrated to act in both phases of enhancer maturation, binding and recruiting on DNA specific chromatin modifiers [20].

All these findings indicate that the dynamic regulation of gene expression throughout organ development and maturation reflects the coordinated activity of chromatin remodeling and transcription factor/enhancer interactions, which are precisely tuned to tissue context and differentiation state.

While HNF4α differently binds to and remodels chromatin on different genes, allowing tissue-specific transcription, it can also drive similar gene expression programs in different adult tissues that share common functions. For example, HNF4α has been shown to regulate the brush border gene program across multiple epithelial tissues, including the intestine, kidney, and yolk sac, where it maintains epithelial identity and chromatin organization underlying brush border specialization [1]. Loss of HNF4α in these organs disrupts brush border gene expression and compromises epithelial transport functions. ChIP-seq analyses revealed that HNF4α directly binds and activates brush border genes, whereas H3K4me3 HiChIP-seq demonstrated that its loss abolishes chromatin loops connecting enhancers and promoters of these genes, leading instead to enhanced looping at stress fiber loci. Interestingly, the interaction of HNF4α with the enhancer-looping factor LDB1 has been described in hepatocytes, where is required for the regulation of hundreds of liver metabolic genes through enhancer–promoter interactions that correlate with histone acetylation (i.e., H3K27ac) and transcription [43].

## 5. HNF4α-Driven Chromatin Modification and Remodeling

Numerous studies have documented HNF4α binding at active enhancers characterized by specific histone and DNA modifications.

Specific chromatin structures and chemical modifications observed in genomic loci occupied by HNF4α depend on the ability of the transcription factor to recruit specific chromatin modifiers.

Genome-wide studies indicate that HNF4α recruitment to active enhancers drives key modifications, including 5-hydroxymethylcytosine (5hmC) and H3K27 acetylation (H3K27ac) [36]. In hepatocyte-specific Hnf4α knockout mice, these marks were reduced at HNF4α-bound regions. Mechanistically, HNF4α-dependent 5hmC relies on its interaction with the methylcytosine dioxygenase TET3, whose expression in the liver is also directly regulated by HNF4α through enhancer binding [36]. Additionally, HNF4α was shown to interact with the MLL4 methyltransferase complex to facilitate its recruitment to HNF4α-bound regions together with other histone-modifying enzymes, including p300, leading to the enrichment of active histone marks such as H3K4me1 and H3K27ac at transcriptional regulatory regions [20].

Notably, in the HNF4α-dependent transcriptional inhibition, the recruitment of SMRT/NcoR-HDAC complexes plays a role in modulating chromatin structure. The complex, indeed, functions as a transcriptional corepressor, driving gene silencing [44]. As highlighted in studies by Ungaro et al. [45] and Santangelo et al. [9], HNF4α can interact with SMRT/NCoR and drive the complex on target genes to silence their expression [9,45].

Global and gene-specific analysis of histone modifications and DNA methylation in liver revealed that Hnf4α loss in KO mice profoundly impacts histone methylation and acetylation, increasing H3K4me2, H3K4me3, H3K9me2, H3K27me3, and H3K4ac levels, along with elevated expression of histone variants (H1.2, H3.3) and key epigenetic modifiers, including DNMT1, TET3, and histone methyltransferases (SETD7 KMT2C, EHMT2 and EZH2) [46].

Other than its direct roles in epigenetic regulation through the recruitment of chromatin modifiers and the modulation of chromatin loop dynamics, HNF4α also shapes the epigenetic landscape by controlling the expression of chromatin-remodeling factors such as Smarcd3 and the chromatin licensing factor Cdt1, whose expression is lost in HNF4α-deficient livers [47].

HNF4α plays a central role in modulating chromatin accessibility, thereby facilitating the binding of other transcription factors.

In the liver, a recent study highlighted HNF4α’s critical role in shaping the genome-wide binding landscape of the ubiquitously expressed nuclear receptor, the glucocorticoid receptor (GR). Using liver-specific HNF4α knockout models, the authors demonstrated remodeling of chromatin accessibility at GR binding sites, accompanied by a redistribution of GR recruitment [48].

Qu and colleagues [49] further showed that hepatocyte-specific loss of HNF4α in mice markedly reduces genome-wide binding of the circadian master regulators BMAL1 and CLOCK at E-box elements. This was accompanied by decreased levels of epigenetic marks indicative of open chromatin, including H3K4me1 and H3K27ac [49]. Consistently, HNF4α recruitment to DNA, chromatin accessibility, and circadian gene expression exhibited dynamic changes throughout the day, which are disrupted in HNF4α-deficient livers.

Studies on liver regeneration further highlight HNF4α’s central role in chromatin remodeling. Using an integrated approach combining ATAC-seq, TRAP-seq and ChIP-seq in quiescent versus regenerating livers, Wang and colleagues [50] observed that HNF4α levels are reduced in repopulating hepatocytes, leading to reduced HNF4α occupancy at hundreds of genomic sites (primarily liver-specific enhancers) and downregulation of numerous metabolic and biosynthetic genes. At the same time, chromatin accessibility increases at regulatory regions of proliferation-associated genes, favoring the occupancy by transcription factor CTCF. These coordinated epigenetic changes drive the transcriptional reprogramming required for hepatocyte proliferation and effective liver regeneration.

## 6. HNF4α-Dependent Transcription of Epigenetic Non-Coding RNAs

Beyond its well-established role in regulating the expression of epithelial protein-coding genes (including epigenetic modifiers, as discussed above), mounting evidence indicates that HNF4α orchestrates a complex network of non-coding RNAs to maintain epithelial identity and prevent the EMT. Notably, HNF4α can directly regulate some microRNAs, belonging to the subclass of the so-called “epi-miRNAs” [51]. These “epigenetic” miRNAs, by targeting epigenetic, epitranscriptomic and epiproteomic modifiers, orchestrate gene expression and, when deregulated, contribute to cancer and other pathological conditions.

HNF4α directly induces the transcription of miR-29a/b, which suppresses the de novo DNA methyltransferases DNMT3A and DNMT3B [52,53]. As shown by Cicchini and coworkers, the rearrangement of DNA methylation pattern is needed for the acquisition of mesenchymal properties by the hepatocytes undergoing EMT [52]; therefore, through a miR-29a/b-mediated mechanism, HNF4α couples transcriptional and epigenetic regulation to maintain the epithelial phenotype. Interestingly, miR-29a has also been reported to upregulate the Hippo pathway through the relief of DNMT3B-mediated LATS1 methylation in HCC, thus interfering with YAP expression [53], and potentially with the YAP-mediated HNF4α downregulation [54].

In hepatocytes, HNF4α drives the transcription of the miR-194/192 cluster, a well-characterized set of epithelial regulators. Hepatocyte-specific ablation of HNF4α in mice results in a severe decrease in their expression accompanied by reactivation of genes involved in cytoskeletal remodeling, migration, and metabolic reprogramming, thus promoting a partial dedifferentiation. Interestingly, miR-194 targets the histone H3 methyltransferase SETD5 [55] and a member of the Cullin 4B-Ring E3 ligase CUL4B [56] that controls protein epimodifications and whose over-expression is linked to HCC progression and poor prognosis [55,57,58]. In breast cancer, miR-194 also acts as an epi-miRNA by targeting DNMT3A [59], while miR-194-3p controls the expression of the DNA methylation reader MeCP2 [60], allowing the recruitment of DNTMs and histone deacetylases [61].

HNF4α further impacts the epigenome by controlling the expression of miR-101 and miR-193 in hepatocytes [62]. MiR-101 acts as tumor suppressor in lung and breast cancer, by negatively regulating DNMT3A [63] and EZH2 [64]; miR-193 targets DNMT3A and HDAC in acute myeloid leukemia cells [65].

HNF4α is also a direct positive regulator of miR-122 [66], which can be considered an epi-miRNA targeting the histone methyltransferase G9a [67] and the SWI/SNF subunit SMARCD1/BAF60a [68]. While G9a promotes HCC progression, SMARCD1/BAF60a is required for the coordinated expression of metabolic pathways [69], highlighting the fine-tuned modulation of different targets.

Another HNF4α-induced epi-miRNA with tumor suppressive activity in HCC progression is miR-124 [70]. Interestingly, miR-124 limits colorectal cancer progression by negatively regulating DNMT3B and DNMT1 [71]. Additionally, miR-124-3p.1 targets the lysine deacetylase Sirtuin 1 (SIRT1) in HCC [72] and DNMT3B in bladder cancer [73].

Nuclear long non-coding RNAs (lncRNA) represent a further subclass of ncRNAs with a key role as epigenetic regulators of transcription recruiting chromatin remodeling factors and modifiers [74]. In analogy with epi-miRNAs they can be referred to as “epi-lncRNAs”.

The oncogenic lncRNA HOTAIR (HOX Transcript Antisense Intergenic RNA), a central player of the EMT molecular mechanism, is a well-characterized negative transcriptional target of HNF4α belonging to this category. HOTAIR organizes molecular platforms including epigenetic modifiers that are recruited to specific genomic loci inducing chromatin remodeling and transcriptional regulation. Mechanistically, HOTAIR functions as a scaffold for the Polycomb Repressive Complex 2 (PRC2, including the H3K27 methyltransferase EZH2, SUZ12, and EED), promoting H3K27 trimethylation and gene silencing [75]. The genome-wide PRC2 retargeting drives a broad transcriptional reprogramming associated with EMT and increased invasiveness [76]. Coherently, HOTAIR is widely dysregulated in cancer and emerged in the last few years as a key regulator of epithelial plasticity and EMT/MET dynamics [77,78,79,80]. While HOTAIR expression is stably down-regulated by HNF4α in several models of epithelial cells, including hepatocytes, its expression is reactivated during EMT/MET and in hepatocyte-specific Hnf4a knockout mice and colon cancer cells [81]. Mechanistically, HNF4α exerts this repression by reshaping local chromatin topology, through the disruption of a functional loop between a distal enhancer and the proximal promoter of HOTAIR [81]. Notably, TGF-β1 functionally inactivates and transcriptionally represses HNF4α [82], thereby relieving its inhibitory control over HOTAIR and enabling the transcriptional reprogramming that drives EMT [81]. Specific microRNAs are involved in this regulatory axis. HOTAIR sponges the epi-miR-29b [83]). miR-34a [84,85] and miR-200c [86], up-regulated by HNF4α in differentiated hepatocytes [87] and established suppressors of EMT and tumorigenesis [88,89], directly target HOTAIR. MiR-34a and miR-200c can also act as tumor suppressors by directly regulating EZH2 levels in breast and in lung cancers, respectively [88,90].

Furthermore, the lncRNA H19 has also been identified as an HNF4α-target gene involved in liver metabolic zonation [91]. H19 can be considered an epi-lncRNA, as it can recruit or upregulate chromatin modifier, such as the histone methyltransferase EZH2 [92], or regulate chromatin readers like HP1 through miRNA-mediated mechanisms (i.e., miR-675) [93], although these mechanisms have been described in cancer contexts so far.

Overall, these findings depict complex circuits where HNF4α-driven epi-ncRNAs sustain epithelial identity and restrain mesenchymal programs, by coupling transcriptional and post-transcriptional control.

Of note, some epithelial lncRNAs serve as host genes for HNF4α-dependent miRNAs, thus tightly linking multiple layers of regulation by HNF4α through different types of ncRNAs. For instance, the epi-miR-192/miR-194 cluster resides within the lncRNA MIR194-2HG, directly transcribed by HNF4α [94,95]; the loss or inactivation of HNF4α reduces host gene transcription and consequently miRNA levels. Therefore, HNF4α can regulate microRNA expression both through direct promoter binding but also indirectly via host genes.

Through these mechanisms, HNFα integrates transcriptional regulation with miRNA biogenesis, enabling precise control of liver-specific miRNA networks and broader post-transcriptional gene regulation, ultimately impacting the epigenetic landscape.

## 7. Dysregulation of HNF4α-Driven Epigenetic Reprogramming in Epithelial Diseases

The loss of HNF4α expression and function has been closely linked to the progression of a wide range of chronic human diseases. Its dysregulation contributes not only to metabolic and developmental disorders, such as Type-2 diabetes, MODY1, and non-alcoholic fatty liver disease (NAFLD/NASH), but also to inflammatory conditions, including inflammatory bowel disease (IBD) as well as chronic and viral hepatitis [13,29,96]. Furthermore, HNF4α impairment is implicated in the development of organ fibrosis (i.e., liver and renal fibrosis) [97,98] and in the onset of various cancers, including hepatocellular carcinoma, renal cell carcinoma, and pancreatic ductal adenocarcinoma [99,100]. In recent years, a significant role of HNF4α in the pathogenesis of various hematological disorders has also been recognized, such as myelodysplastic syndromes, acute myelogenous leukemia (AML), and anemia associated with metabolic or inflammatory disorders (reviewed in [101]).

Given the established role of HNF4α as an epigenetic regulator, it is likely that its dysfunction contributes to disease progression not only through conventional transcriptional control but also through epigenetic reprogramming. This reprogramming can alter chromatin landscapes and gene expression patterns, promoting metabolic dysfunction, inflammation, fibrogenesis, and tumorigenesis. Accordingly, in many diseases where the expression or activity of HNF4α is dysregulated, epigenetic reprogramming plays a key role in driving the pathological process [102,103,104], although only limited evidence demonstrates a direct involvement of HNF4α.

During inflammatory stress, it has been observed that the downregulation of HNF4α is required for full activation of acute-phase genes. Conversely, its sustained expression actively suppresses the inflammatory response, protecting hepatic identity and functional integrity under stress conditions [105]. In particular, epigenetic analyses using FAIRE qPCR and ChIP qPCR in a liver-on-chip organoid model as well as in co-cultures of hepatocytes with endothelial cells and macrophages (mimicking a physiologically relevant tissue environment), revealed that regulatory regions of acute-phase genes remain in a closed chromatin state when HNF4α is overexpressed and directly bound to inflammation-related acute-phase genes. This regulation is lost upon pro-inflammatory stimuli. These findings position HNF4α as a gatekeeper of the liver homeostasis, which can be compromised in inflammation-related liver pathologies such as hepatitis, metabolic diseases and cirrhosis [13].

Further studies showed that HNF4α controls gluconeogenesis, thus possibly impacting on the onset of type 2 diabetes (T2D), through the direct recruitment to specific genome sites of TET2 [106], thus leading to DNA demethylation and gene expression regulation [107]. TET2 is recruited by HNF4α on the Fructose 1,6-bisphosphatase (FBP1) promoter, inducing its expression in response to glucagon stimulation [106].

Chahar and colleagues [108] profiled genome-wide histone modifications in a model of colon inflammation, where a downregulation of HNF4α is observed. They found a significant decrease in H3K27ac specifically in loci enriched for HNF4α binding sites and a parallel increase in occupancy by inflammatory factors such as AP-1 and NF-κB that determine an epigenetic switch from epithelial identity to an inflammatory program. The enhancer repositioning has been directly associated with the pathogenesis of IBD and the risk of neoplastic progression in models of chronic colitis. Notably, immune regulatory genes were identified as direct transcriptional targets of HNF4α, suggesting that HNF4α restoration could be a strategy to counteract acute colitis with potential relevance for IBD [108].

Loss of HNF4α may also promote tumor-associated inflammation through a microRNA-inflammatory feedback loop circuit. One of the most characterized examples of such regulation is the oncogenic feedback loop described in HCC where HNF4α induces the transcription of miR-124, which in turn attenuates IL-6R/STAT3 signaling. Activated STAT3, instead, enhances the expression of miR-24 and miR-629, two miRNAs that directly repress HNF4α, thus reinforcing its silencing [70]. HNF4α also engages in a second, miRNA-mediated feedback loop, involving NF-κB. In this circuit, HNF4α upregulates miR-124 and miR-7, which repress RelA (p65), whereas NF-κB drives expression of miR-21 that suppresses HNF4α, amplifying the inflammatory response [109]. Notably, a key player in both circuits is miR-124, which, as discussed above, can suppress tumorigenesis by targeting the DNA methyltransferases DNMT1 and DNMT3B [71,73] as well as the deacetylase SIRT1 [72]. Interestingly, a pharmacological inhibitor of DNMT1 has been proposed for the treatment of HCC [110].

## 8. Conclusions

Cumulative and direct experimental evidence presented in this review, positions HNF4α as a primary driver of genome-wide transcriptional and epigenetic reprogramming. This regulation is achieved through the coordinated activity of: (i) chromatin remodelers that enable the dynamic recruitment of transcription factors and epigenetic modifiers, (ii) chromatin-modifying enzymes, including DNMTs and DNA demethylases, HDACs and HATs, as well as histone methyltransferases and demethylases, (iii) structural cofactors and chromatin-organizing proteins that shape local chromatin architecture by establishing specific enhancer–promoter loops, and (iv) epigenetic regulatory non-coding RNAs (Table 1). Collectively, these multilayered HNF4α-centered regulatory networks maintain epithelial identity, suppress dedifferentiation and EMT, and restrain tumor progression. Conversely, the loss of HNF4α expression and function (due to inflammation, oncogenic signaling, extracellular cues or loss of epithelial identity), contributes to pathological states, highlighting that the restoration of HNF4α-driven epigenetic regulations may represent a promising strategy for therapeutic intervention, particularly for cancer, where HNF4α-mediated regulation of epithelial identity and EMT is frequently disrupted.

Notably, even if the HNF4α-driven networks of epi-ncRNAs and chromatin modifiers have been well-documented, further studies are needed to establish the direct functional existence of these regulatory axes and their role in specific physiological and pathological contexts. A deeper understanding of how HNF4α orchestrates these complex interactions will be essential to translate this knowledge in therapeutic strategies.

## Figures and Tables

**Table 1:** HNF4α-Dependent Epigenetic Regulations.

Epigenetic Regulations	HNF4α-Mediated Epigenetic Mechanism	HNF4α-Driven Epigenetic Output
Chromatin remodeling	Recruitment of chromatin remodeling factors (SWI/SNF-BAF) ^(a)^ [41]. Transcriptional control of chromatin remodeling SMARCD3 ^(b)^ [47]. Interaction with enhancer looping factor LDB1^(b)^ [43] and with cohesin components ^(a)^ [41]. Change of 3D chromatin structure (loop disassembly) restraining HOTAIR expression ^(c)^ [81].	Maintenance of chromatin accessibility Chromatin looping dynamics
Histone Modifications	Recruitment of MLL4 (H3K4me1) and p300/CBP (H3K27Ac) ^(b)^ [20,36]. Transcriptional down-regulation of histone methyltransferases ^(b)^ [46]. Recruitment of the SMRT/NCoR-HDAC complex ^(b,d)^ [9,45]. Epi-ncRNA-mediated regulation of HDACs and histone methyltransferases (see below)	Induction and stabilization of permissive epigenetic state of regulatory regions Restriction of pro-EMT histone acetylation
DNA Methylation	Interaction with and transcriptional regulation of TET3 (5hmC) ^(b)^ [36,46] and TET2 (5hmC) ^(d)^ [106] Transcriptional down-regulation of DNMT1 ^(b)^ [46]. Epi-ncRNA-mediated regulation of DNMTs and DNA readers (see below)	Reversion of non-permissive epigenetic state of regulatory regions. Restriction of pro-EMT methylation
epi-ncRNA regulation	miR-29a/b [52] targeting DNMT3A and, DNMT3B [52,53] miR-194 [55] targeting the histone-H3-methyltransferase SETD5 [55], the DNA reader methylation MeCP2 [60], the epi-protein modifier Cul4B [56] and DNMT3A [59]. miR-122 [66] targeting histone methyltransferase G9A [67] and chromatin remodeling complex subunit SMARCD1/BAF60A [68]. miR-124 [70] targeting lysine deacetylase SIRT1 [72], DNMT1 and DNMT3B [71,73] miR-101 [62] targeting DNMT3A [63,65] and EZH2 [64]. miR-193 [62] targeting DNMT3 and HDAC [65] miR-200 [87] targeting EZH2 [88] miR-34 [87] targeting EZH2 [90] lncRNA H19 [91] required for post-differentiation of hepatocytes [91], targeting EZH2 [92] and the chromatin reader HP1 through miRNA regulation [93]. lncRNA MIR-194-2HG, host gene of the miR-194/192 cluster [94], targeting DNMT3A [59]. lncRNA HOTAIR [81] required for EZH2 recruitment [75,80].	Up- and down-regulation of epithelial and mesenchymal programs by: transcriptional upregulation of epithelial and anti-EMT epi-miRNAs transcriptional up-regulation of epi-lncRNAs transcriptional down-regulation of pro-EMT epi-lncRNAs

^(a)^ Intestinal epithelium cells, ^(b)^ hepatocytes/liver cells, ^(c)^ colon cancer cells, ^(d)^ hepatocellular carcinoma cells.

## Data Availability

No new data were created or analyzed in this study.

## Abbreviations

The following abbreviations are used in this manuscript:

HNF4	Hepatocyte nuclear factor
EMT	Epithelial-to-mesenchymal transition
MET	Mesenchymal-to-epithelial transition
HCC	Hepatocellular Carcinoma
PRC2	Polycomb Repressive Complex 2
HOTAIR	HOX Transcript Antisense Intergenic RNA
SIRT1	Sirtuin 1
lncRNA	long non-coding RNA
IBD	Inflammatory bowel disease
